# An Evaluation of the Sodium Content and Compliance with the National Sodium Reduction Targets among Packaged Foods Sold in Costa Rica in 2015 and 2018

**DOI:** 10.3390/nu11092226

**Published:** 2019-09-15

**Authors:** Jaritza Vega-Solano, Adriana Blanco-Metzler, Karla Francela Benavides-Aguilar, JoAnne Arcand

**Affiliations:** 1 Costa Rican Institute of Research and Teaching in Nutrition and Health (INCIENSA), Tres Ríos Box 4-2250, Costa Rica; 2 Faculty of Health Sciences, Ontario Tech University (University of Ontario Institute of Technology), 2000 Simcoe St N, Oshawa, ON L1H 7K4, Canada

**Keywords:** salt, sodium, sodium reduction, sodium targets, process food, food industry, food policy, Costa Rica, Latin America

## Abstract

High blood pressure is a leading cause of death in Costa Rica, with an estimated mortality rate of 30%. The average household sodium intake is two times higher than the World Health Organization recommendation. The consumption of processed foods is an important and growing contributor to sodium intake. The objective of this study was to describe the sodium content of packaged foods (mg/100 g) sold in Costa Rica in 2015 (*n* = 1158) and 2018 (*n* = 1016) and to assess their compliance with the national sodium reduction targets. All 6 categories with national targets were analyzed: condiments, cookies and biscuits, bread products, processed meats, bakery products, and sauces. A significant reduction in mean sodium content was found in only 3 of the 19 subcategories (cakes, tomato-based sauces, and tomato paste). No subcategories had statistically significant increases in mean sodium levels, but seasonings for sides/mains, ham, and sausage categories were at least 15% higher in sodium. Compliance with the national sodium targets among all foods increased from 80% in 2015 to 87% in 2018. The results demonstrate that it is feasible to reduce the sodium content in packaged foods in Costa Rica, but more work is needed to continually support a gradual reduction of sodium in packaged foods, including more stringent sodium targets.

## 1. Introduction

Excess dietary sodium causes high blood pressure (HBP) and is associated with an increased risk of hypertension and cardiovascular disease (CVD) in both men and women [1,2]. CVDs are the primary cause of death globally, resulting in over 15 million deaths in 2015 [3,4], with HBP as the main risk factor [5,6]. Since the 1970s, HBP has been the leading cause of death in Costa Rica, with an estimated mortality rate of 30% [7]. In 2014, the prevalence of HBP in Costa Rica was 36.2%, in adults >19 years of age, exceeding 60% in those >65 years [8]. In 2012, the cost of healthcare for HBP represented 3.47% of Costa Rica’s Social Security expense alone [9]. 

The Household Budget Survey (HBS) administered in Costa Rica during the periods of 2004–2005 and 2012–2013 found that the population sodium intakes are an estimated 3900 and 4600 mg/person/day, respectively. These levels far exceed the dietary sodium recommendations by the World Health Organization (WHO) of 2000 mg per day (5 g of salt per day) [10,11]. Dietary sources of sodium in Costa Rica are common salt (60%) and packaged food and condiments (27%), with the remainder coming from foods that naturally contain sodium; however, there is a substantive increasing trend in the amount of dietary sodium derived from packaged foods [10]. 

To address these health and dietary challenges, dietary sodium reduction has become a public health priority. Sodium reduction initatives are recognized by the World Health Organization and Pan American Health Organization (PAHO/WHO) as cost-effective, “best-buy” interventions that have significant potential to impact HBP and CVD outcomes [12,13]. Therefore, worldwide, numerous countries have implemented national sodium reduction strategies [14]. These strategies usually include multiple components, such as the development of sodium reduction targets for packaged and/or restaurant foods, consumer education, front-of-pack nutritional labeling schemes, warning labels, taxes for high sodium foods, and interventions and standards for foods served in public institutions, among others [15,16,17,18,19]. 

In 2010, the Costa Rican Ministry of Health committed to implementing the “Cardiovascular Disease Prevention Initiative through the Reduction of Salt Intake in the Americas,” launched in 2009 by PAHO/WHO, with a goal of gradually reducing sodium to 2000 mg/person/day sodium by 2020 [16]. In 2011, the “National Plan for Salt/Sodium Intake Reduction in Costa Rica’s Population 2011-2021” was created [20], and in 2013 the “National Program for Salt/Sodium Intake Reduction in Costa Rica’s Population" was declared of public and national interest [21]. Subsequently, a goal to reduce sodium intake by 15% and to reduce the prevalence of HBP by 25% was established as part the “National Strategy for the Integral Approach to Chronic Non-Communicable Disease and Obesity, 2013–2021,” under the National Plan [22]. A key component of this plan is to reduce sodium in the food supply, as an impactful strategy to support the Costa Rican population in achieving the dietary sodium recommendations. 

Food supply interventions to reduce population sodium intake requires monitoring and surveillance. Having data on the sodium content of packaged food sold in Costa Rica also allows for the creation of national reduction targets to promote reformulation.

In 2013, the health and food industry sectors in Costa Rica began negotiations that led to the execution of the first public–private partnership between the Ministry of Health and the Costa Rican Chamber of Food Industry. This partnership sought to join efforts and work together in the implementation of strategies to reduce the sodium content of packaged and restaurant foods [23]. In 2016, national voluntary sodium reduction targets were established for six categories and twenty-three subcategories of packaged food that contributed the greatest amounts of sodium to the Costa Rican diet. These targets were voluntary from March 2016 to January 2018 [23]. However, it is unknown if changes in the sodium content of packaged foods have occurred during this period of time. Therefore, the objective of this study was to describe the changes in the sodium content in packaged foods sold in Costa Rica in 2015 and 2018 and to assess their compliance with the national sodium reduction targets.

## 2. Materials and Methods 

### 2.1. Study Design

This study is an analysis of two cross-sectional databases containing food label information on of packaged food sold in 2015 and 2018. The databases contained data on foods that were systematically collected from a large supermarket chains in the Greater Metropolitan Area of the country [24,25]. Data were collected from one supermarket in 2015 and two supermarkets in 2018.

### 2.2. Data Collection

A team of two researchers collected data between June and December 2015 and between January and August 2018. In 2015, collection was carried out through a smartphone application developed by the George Institute for Global Health of Australia [26,27]. In 2018, data were collected with the FLIP-LAC system (Food Label Information Program for Latin America and the Caribbean), developed by the Department of Nutritional Sciences from the University of Toronto and adopted and validated in an International Development Research Centre of Canada-funded research project (#108167) [28]. Regardless of the data capturing software, the sampling framework and procedures were consistent during the two time periods. In both surveys, the barcode of all packaged products was scanned (excluding alcoholic beverages). Photos of all sides of the package were taken to capture required information such as name, brand, ingredients list, serving size, and nutritional information per serving and/or 100 g or 100 mL. In both years, only the information from medium-sized packages was included for analysis. Where foods required nutrition information to be presented “as consumed”, the Institute of Central America and Panama (INCAP) nutrient database was used to create recipes [29]. Food categories that contained items requiring recipes were bouillon cubes and powders and cakes. Quality assurance measures were executed to ensure accuracy and consistency of the data and food classifications. 

### 2.3. Food Classification and Data Analysis

This study captured data on all foods that within food categories and subcategories that aligned with the national sodium reduction targets. Included foods had the sodium content reported on the nutritional label. The list of categories and subcategories with their respective baseline and national target is shown in Table S1. All 6 categories and 19 out of 23 subcategories with national targets were analyzed. The “bread with cheese” and “semi-sweet bread” categories were excluded, because these products are not considered packaged food according to Costa Rican legislation and many do not carry nutrition labels [30,31]. In the bakery category, the “fermented dough without filling” and “unfermented dough” subcategories were excluded because a list of ingredients was not available on the package to verify their subclassification. 

The average, median, standard deviation, minimum, and maximum amount of sodium per 100 g of food in each food category and subcategory were determined. A comparison of the mean sodium content between 2015 and 2018 was assessed using an unpaired *t*-test. The prevalence of compliance with the national sodium targets for each food subcategory was calculated. Categorical data are presented as frequency and percentages. Continuous data are presented as means and standard deviations. A *p*-value < 0.05 was considered statistically significant. Data were analyzed with SPSS version 21 (IBM Corp, Chicago, IL, USA).

## 3. Results

In 2015 and 2018, information on 1158 and 1016 food products, respectively, was collected. Most foods packages contained nutritional labeling information 76% (880 products) in 2015 and 87% (884 products) in 2018. Finally, only products that included information on sodium as part of the nutrition labels were included in the analysis: 724 foods (82%) in 2015 and 791 foods (89%) in 2018. In 2015 and 2018, cookies and biscuits was the food category with the highest number of products that contained labelling on sodium (*n* = 332 and *n* = 355, respectively), followed by sauces (*n* = 129 and *n* = 150), bakery (*n* = 79 and *n* = 58), bread products (*n* = 69 and *n* = 90), processed meats (*n* = 60 and *n* = 79), and condiments (*n* = 55 and *n* = 57) (Table 1). In all categories, a higher number of products were available 2018 compared to 2015.

### 3.1. Sodium Content by Category and Subcategory of Packaged Food from 2015 to 2018

The condiments subcategories had the highest average sodium content, with up to 19,044 mg/100 g as observed with bouillon cubes and powders. The bread subcategories had the lowest sodium content, with sweet and whole grain breads containing 166 and 291 mg/100 g, respectively (Table 1).

On examination of the distribution of sodium levels between 2015 and 2018, 16% of food categories had statistically significant decreases in sodium; thus, 84% had no significant changes in sodium. Statistically significant changes from 2015 to 2018 occurred in tomato-based sauces (mean ± SD, mg/100 g; 920 ± 1030 to 462 ± 265; 50% reduction, *p* = 0.000), tomate paste (417 ± 187 to 231 ± 190; 45% reduction, *p* = 0.007), and cakes (452 ± 272 to 341 ± 240; 25% reduction, *p* = 0.012). Median sodium levels showed declining trend in most subcategories. 

A high degree of variability in the sodium content within the food categories and subcategories was observed in both years. In particular, the highest variance was observed among meat and fish seasonings, dry salted crackers, English sauce, and tomato sauce. Extreme values were found in most subcategories (Table 1). A lower variability in sodium levels was observed in 2018, compared to 2015, for some categories such as bread and sauces. Variation in the condiments and cookies and biscuits sub categories remained similar between 2015 and 2018, and there was greater homogeneity (Table 1). Variability was similar in subcategories with the lowest number of food products (condiments for side and other main dishes, filled salted crackers, sweet bread, *salchichón*, and *mortadella*) in both years, except for the *salchichón* subcategory, where the limits increased widely due to a small sample size (Table 1).

### 3.2. Compliance with National Targets for the Sodium Reduction in Packaged Food by Category and Subcategory

**Figure 1:**
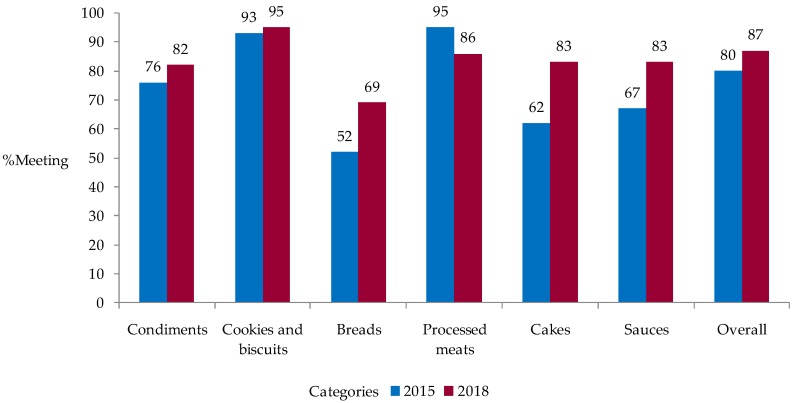
Comparison between years of the compliance with the national target for the sodium reduction in packaged food categories, Costa Rica, 2015–2018.

There was slight increase in the proportion of foods that met the national targets from 80% in 2015 to 87% in 2018 (Table 2, Figure 1). In 2018, cookies and biscuits had the highest level of compliance (95%), while breads had the lowest level of compliance (69%) (Table 2, Figure 1). With the exception of processed meats, all the categories had an increased proportion of foods that met the national targets (Figure 1). The greatest improvements were observed among cakes, bread and sauces with a 21%, 17% and 16% percentage point increase, respectively. Improvements in the compliance with the national targets was consistent with changes to mean sodium levels observed in the earlier analysis.

Among subcategories, the greatest positive changes in meeting the national targets were seen among sweet bread (67% to 100%), wholemeal bread (50% to 82%), tomato-based sauces (59% to 81%), English sauce (68% to 86%), bouillon cubes and powders (33% to 50%) and cakes (62% to 83%). Three categories had a lower proportion meeting the national targets: Sausages (100% to 71%) *Salchichón* (100% to 33%) and ketchup (81% to 74%). There was no change observed among seasonings for side and main dishes, dry sweet cookies, mortadella, and ham.

On examination of the 2018 data alone, overall, there was a high level of compliance. Sweet bread, mortadella, as well as seasoning for side and main dishes achieved 100% compliance (Table 2). Only two subcategories had a compliance of 50% or less: bouillon, cubes and powders (50%), as well as *salchichón* (33%).

## 4. Discussion

This is the first longitudinal study performed in Costa Rica regarding the changes to sodium levels in packaged food, before and after the implementation of national targets. Costa Rica is the first Central American country to implement a national plan for the reduction of sodium intake [32]. This was achieved by commitment and dedication from both the Ministry of Health and Costa Rican Chamber of Food Industry in establishing a private–public partnership in 2014 to reduce sodium in processed food, which included the development and adoption of voluntary targets for the reduction of sodium in packaged in 2016, with a renewal in 2019 [33]. 

The implementation of sodium reduction targets requires the food industry to reformulate products, which represents a challenge in certain foods due to functional properties of sodium, such as the provision of microbiological protection, its influence in taste and texture, and being a preservative, among others [34]. However, there is evidence that its progressive and gradual reduction is feasible and allows the food industry to develop alternatives for a more significant reduction and to ensure the adaptation of consumers of food with less sodium [35].

Between 2015 and 2018, 84% of food subcategories analyzed did not have a statistically significant reduction in mean sodium content. However, while not all data were statistically significant, there were some meaningful changes that show some improvement, which may have resulted from efforts within Costa Rica or regionally. For example, the cakes subcategory which had significantly lower sodium in 2018, also has a regional sodium target that was established by the Salt Smart consortium; thus, this decrease may be linked to food industry efforts at the regional level [36]. In the case of the other two subcategories, the tomato-based sauces and the tomato paste, the main food production industries are also transnational companies recognized for initiating sodium reduction processes in their products in parallel with the establishment of regional goals [37,38,39,40].

Furthermore, the analysis found a high variability of sodium content within the same subcategory, but in some categories, this variance decreased over time. This wide variability in a same subcategory establishes the feasibility of reformulating foods that have a higher sodium content.

In 2018, a larger sample size was found in some subcategories that had meaningful but nonsignificant increases in sodium content (seasonings for side and main dishes, sausages, *salchichón*, and ham). Since the same methodology was used to systematically sample data in 2015 and 2018, this larger sample could be the result of the emergence of new products on the market that are higher in sodium. The results may show the influence of a dynamic food marketplace, characterized by a broad rotation of new products that mostly contain more sodium, directly affecting the mean and median sodium levels as well as variation observed. Future research should assess the sodium levels and compliance of these foods alone, and impact on population sodium intake. 

The actions taken by the food industry to introduce a variety of lower sodium products are recognized, but it remains necessary to continue to increase their availability and accessibility to the consumer [33]. These actions are reflected in the current study, which found that the majority of foods offered in 2015 (80%) already contained sodium levels that were below the national sodium reduction targets, a compliance rate that increased by 7% in 2018. In addition, it is evident that: (1) It is feasible for the food industry in Costa Rica to reduce the sodium content of the foods they produce, and (2) there needs to be a progressive and continuous effort to reduce sodium among all of the sectors involved, and the successes and challenges of these efforts should be monitored. For many of the large food companies with sodium reduction programs, voluntary targets as a first step may not require significant efforts. Although food reformulation is feasible [14], sodium reduction efforts should be periodically reassessed, following the example of countries such as the United Kingdom, Canada, and South Africa [15,41,42].

The results obtained are consistent with those of other countries, whether the sodium reduction targets were voluntary or mandatory [17,35,43,44,45,46]. For example, in Argentina, several food categories had a high proportion of products below the voluntary and mandatory targets in 2011 and 2014, respectively [35]. Brazil has had a voluntary sodium reduction plan since 2011. An evaluation of compliance in 2017 revealed that most of the categories already met the national target and even the regional targets. Additionally, most of the categories achieved a mean reduction between 8% and 34%, resulting in the need to implement more stringent targets [17,45]. In Canada, 51.4% of packaged food already met the sodium value set in the national and voluntary targets [43]. A recent analysis carried out between 2012 and 2016 showed no progress in the compliance of 48% of the products [44]. Moreover, the 2015 baseline study performed in fourteen countries of Latin America and the Caribbean, including Costa Rica, met an 82% general compliance with the regional targets. Costa Rica stands out for submitting one of the lowest proportions of food (77%) that met regional targets [46]. 

Although the compliance rate in Costa Rica and Latin America are high, sodium intakes continue to exceed recommended intakes in Costa Rica and LAC; therefore, there is a need to consider adopting stricter targets for optimal public health benefit. Nilson and Combris [17,18] highlighted the need to gradually adjusting national targets over time. The Salt Smart Consortium emphasized the same concept and agreed to updating the regional targets every two years [36]. However, when considering an update to targets, the food industry compliance, the target levels themselves, and the addition of new categories and subcategories must be assessed at a country level. In Costa Rica, the public–private partnership between the Ministry of Health and the Costa Rican Chamber of Food Industry itself indicated the possibility to include other categories of food identified as future priority in the previous version and reiterates it with its renewal. The targets set with biannual gradual reduction until 2022 should also be assessed, considering the international guidelines, the most recent data on the sodium consumption at the population level, the technological viability, and the acceptability of the consumer [33].

The Pan American Health Organization/ World Health Organization (PAHO/WHO) highlights, through the Declaration of the Salt Smart Consortium, that a significant strategy to reduce the salt intake by the entire population requires the cooperation of food processors and importers to also reduce the presence of salt (table salt) in the food supply itself. However, it also suggests significant effort and coordination is required to modify the social norms for the consumers and to engage them in self-regulating the amount of salt they add to food [36]. The data in the current study align with the need to implement other measures for the achievement of the national plan target [20], which includes the development of interventions such as social marketing and communication campaigns, education, the promotion of easy to interpret front-of-pack food labeling, and the involvement of the civilian population in decision-making related to public health, among others.

The study has limitations. Nutritional labelling is not mandatory in Costa Rica; therefore, it is not possible evaluate the compliance in all foods [30,31]. Despite this, the majority of foods do contain labeling and information about sodium, and the results are consistent with similar analyses in other countries [47]. This data also suggest that legislation should follow mandatory inclusion of the sodium content on food labels to allow the evaluation of compliance with the national targets. This is relevant in those categories and subcategories that currently have a smaller proportion of products with labeling. Our analysis is based on the reported information on the nutrition label, and we make an assumption that these reported values are correct. We note that variation may exist between the label and actual sodium content, particularly for certain food categories or among smaller food manufacturers [48], and because in Costa Rica, legislation allows up to ± 20% variance [30,31]. In addition, for some foods, the research team prepared recipes so that the nutritional composition of a food product reflected what would actually be consumed by an individual. While recipes were created according to preparation instructions on the package, we acknowledge that there may be inherent variation in actual recipes used by the population. Finally, some subcategories such as seasonings for side and main dishes, bouillon, cubes and powders, filled salted crackers, sweet bread, *salchichón*, and *mortadella* have little variety of trademarks and product versions that may be due to the small size of the country, the presence of dominant companies monopolizing the market or because of the special characteristics of the products that that are made for a small market. It must also be considered that voluntary policy measures like these require not only industry commitment, but also continuous monitoring by the government to assess the implementation of sodium reduction initiatives and changes in the food nutritional profile [34].

## 5. Conclusions

This study provides the first evaluation of sodium content in packaged food in Costa Rica. A relatively high proportion of foods compy with the national targets levels, suggesting that some food categories need to be made more stringent. Continuous monitoring and updating of national targets by government authorities in collaboration with the food industry and external financial entities is needed. It this regard, it is recommended to establish a program for monitoring and researching the sodium content in processed foods, through analyzing the nutritional labeling and, ideally, chemical analysis. As part of the official monitoring process, periodic reports on changes to the sodium content of food should be made public and shared with food companies. Finally, this future work should be extended to capture actual changes in dietary intake that result from food product reformulation, as well as any changes in clinical outcomes, such as improved rates of hypertension and cardiovascular disease.

## Figures and Tables

**Table 1:** Sodium content in packaged food by subcategories with national reduction targets, Costa Rica 2015–2018.

Subcategory	Year	n	Sodium (mg/100 g)					
			Mean ±SD	Median	Minimum	Maximum	% Difference	*P* Value
Sweet bread	2015	3	447 ± 439	463	0	877	−63	0.399
	2018	3	166 ± 233	66	0	432		
Tomato-based sauces	2015	71	920 ± 1030	543	246	5415	−50 *	0.000
	2018	93	462 ± 265	393	0	1857		
Tomato paste	2015	13	417 ± 187	363	0	814	−45 *	0.007
	2018	31	231 ± 190	197	0	636		
Wholemeal bread	2015	14	435 ± 310	505	0	936	−33	0.139
	2018	33	291 ± 242	291	0	936		
Ketchup	2015	26	922 ± 572	666	337	2045	−26	0.089
	2018	19	685 ± 337	627	140	1187		
Cakes	2015	79	452 ± 272	344	87	1334	−25 *	0.012
	2018	58	341 ± 240	344	0	1108		
*Mortadella*	2015	3	820 ± 338	643	607	1210	−24	0.420
	2018	2	622 ± 75	623	569	675		
Meat and fish seasonings	2015	40	10,946 ± 9762	6000	133	33,636	−22	0.243
	2018	45	8545 ± 8948	5517	167	33,636		
English sauce	2015	19	1927 ± 592	1907	590	2633	−16	0.283
	2018	7	1620 ± 622	1500	700	2633		
Filled salted crackers	2015	9	796 ± 225	793	516	1235	−12	0.527
	2018	6	702 ± 294	552	500	1265		
Dry sweet cookies	2015	144	304 ± 192	287	0	1800	−12	0.086
	2018	161	269 ± 155	267	0	1250		
Filled sweet cookies	2015	120	311 ± 290	275	38	2750	−11	0.411
	2018	112	277 ± 336	250	9	3419		
Dry salted crackers	2015	59	784 ± 242	800	0	1259	−10	0.059
	2018	76	706 ± 227	726	5	1433		
Bread	2015	52	471± 357	483	0	1668	−5	0.670
	2018	54	448± 159	480	14	767		
Bouillon cubes and powders	2015	9	19,044 ± 6666	20,800	7840	27,000	−3.2	0.846
	2018	14	18,441 ± 7886	20,300	1000	26,400		
Sausages	2015	20	874 ± 173	87	500	1214	+15	0.182
	2018	17	1003 ± 353	900	345	1720		
Ham	2015	35	995 ± 443	929	290	2321	+16	0.075
	2018	49	1158 ± 345	1132	325	2321		
Seasonings for side and main dishes	2015	6	12,366 ± 9589	9883	1427	27,000	+32	0.470
	2018	8	16,269 ± 9755	14,622	6000	28,000		
*Salchichón*	2015	1	736	736	736	736	**	NA
	2018	3	5923 ± 8214	1540	830	15,400		

Data presented as mean ± standard deviation. *p*-values for comparison of mean sodium levels 2015 vs. 2018. * Significant (*p* ≤ 0.05); ** Data not assessed due to small sample size.

**Table 2:** Compliance with national target for the sodium reduction in packaged food by categories and subcategories, Costa Rica, 2015–2018.

Subcategory	Sodium National Target (mg/100 g)		% Meeting National Target (n)		
		n	2015	n	2018
Condiments (Overall)		55	76% (42)	67	82% (55)
Seasonings for side and main dishes	33,100	6	100% (6)	8	100% (8)
Meat and fish seasonings	23,000	40	82% (33)	45	88% (40)
Bouillon cubes and powders	20,500	9	33.3% (3)	14	50% (7)
Cookies and Biscuits (Overall)		332	93% (310)	355	95% (337)
Dry salted crackers	1066	59	90% (53)	76	96% (73)
Filled salted crackers	1111	9	88% (8)	6	83% (5)
Dry sweet cookies	485	144	94% (135)	161	94% (152)
Filled sweet cookies	485	120	95% (114)	112	96% (107)
Breads (Overall)		69	52% (36)	90	69% (62)
Bread	500	52	52% (27)	54	59% (32)
Sweet bread	500	3	67% (2)	3	100% (3)
Wholemeal bread	500	14	50% (7)	33	82% (27)
Processed meats (Overall)		60	95% (57)	71	86% (61)
Sausages	1235	20	100% (20)	17	71% (12)
*Salchichón*	1425	1	100% (1)	3	33% (1)
*Mortadella*	1282	3	100% (3)	2	100% (2)
Ham	1805	35	94% (33)	49	94% (46)
Cakes (Overall)	512	79	62% (49)	58	83% (48)
Sauces (Overall)		129	67% (87)	150	83% (124)
English sauce	2250	19	68% (13)	7	86% (6)
Ketchup	990	26	81% (21)	19	74% (14)
Tomato-based sauces	616	71	59% (42)	93	81% (75)
Tomato paste	572	13	85% (11)	31	94% (29)
Overall		724	80% (581)	791	87% (687)

Data presented as % (n). Bouillon cubes and powders and cakes presented “as consumed”.

## Supplementary Materials

The following are available online at https://www.mdpi.com/2072-6643/11/9/2226/s1, Table S1: List of food categories and subcategories with national target for the sodium reduction on January 2018, Costa Rica.
