# Supplementary material for: An Evaluation of the Sodium Content and Compliance with the National Sodium Reduction Targets among Packaged Foods Sold in Costa Rica in 2015 and 2018
Source: Nutrients. 2019 Sep 15;11(9):2226. doi: 10.3390/nu11092226 (PMC6770815; doi:10.3390/nu11092226)
Supplement: Supplementary file 1 [file nutrients-11-02226-s001.pdf]

Table S1. List of food categories and subcategories with national target for the sodium reduction on January 2018, Costa Rica.

Category	Subcategory	National target (mg/100g)
Condiments	Seasonings for side and main dishes	33,100
	Meat and fish seasonings	23,000
	Bouillon cubes and powders	20,500
Cookies and biscuits	Dry salted cracker	1066
	Filled salted cracker	1111
	Dry cookie	485
	Filled cookie	485
Bread products	Bread	500
	Sweet bread	500
	Wholemeal bread	500
Processed meats	Sausages	1235
	Salchichón[1]	1425
	Mortadella[2]	1282
	Ham	1805
Bakery products	Cakes	512
Sauces	English sauce[3]	2250
	Ketchup	990
	Tomato and other vegetables-based sauces	616
	Tomato paste	572

Reference: Alianza Público-Privada Ministerio de Salud y CACIA para implementar acciones y fomentar estilos de vida saludable, asociados particularmente al sodio, que incluyen una alimentación saludable y equilibrada, nutricionalmente adecuada DM-AM-376-2016. [1] *Salchichón*: Cooked product, prepared on a base of fresh or frozen meat, with or without the addition of cooked meat, of authorized animals, with the addition of edible fat, perfectly crushed and mixed, emulsified or not, made with allowed ingredients and introduced in authorized casings with a minimum 113 mm and a maximum 160 mm perimeter, smoked or not. [2] *Mortadella:* Cooked product, prepared on the basis of fresh or frozen meat, with added or not of cooked meat, of authorized animals, with added edible fat, perfectly crushed and mixed, emulsified or not, made with ingredients of permitted use and introduced in covers authorized with a perimeter greater than 160 mm, smoked or not. Reference: Technical regulation RTCR 411:2008 Processed meat products: Sausage, *Salchichón*, Mortadella and *Chorizo*. Available on: http://www.pgrweb.go.cr/scij/Busqueda/Normativa/Normas/nrm_texto_completo.aspx?nValor1=1&nValor2=64990&nValor3=75733. [3]English Sauce: Non-emulsified, Worcestershire type sauce. It is not only used to accompany dishes, but it is also used as a condiment and even to prepare other types of sauces. The liquid and fermented composition of the English type sauce makes it perfect to serve with both cooked and raw foods. It is used in many recipes of the Costa Rican cuisine, as part of the ingredients for the preparation of dishes such as *Gallo Pinto* or served directly on food as a dressing, for example in tamales. Available on: https://extranet.who.int/nutrition/gina/sites/default/files/COMIECO%202011%20Etiquetado%20Nutricional%20de%20Productos%20Alimenticios%20Preenvasados%20para%20Consumo%20Humano.pdf
